# Impact of walk advice alone or in combination with supervised or home-based structured exercise on patient-reported physical function and generic and disease-specific health related quality of life in patients with intermittent claudication, a secondary analysis in a randomized clinical trial

**DOI:** 10.1186/s12955-023-02198-8

**Published:** 2023-10-23

**Authors:** Anna Sandberg, Maria Bäck, Åsa Cider, Lennart Jivegård, Birgitta Sigvant, Joakim Nordanstig

**Affiliations:** 1 Institute of Medicine, Department of Molecular and Clinical Medicine, Sahlgrenska Academy, University of Gothenburg, Gothenburg, Sweden; 2 Department of Occupational Therapy and Physiotherapy, Sahlgrenska University Hospital, Vita Stråket 13, Gothenburg, 413 45 Sweden; 3 Department of Health and Rehabilitation/Physiotherapy, Institute of Neuroscience and Physiology, Sahlgrenska Academy, University of Gothenburg, Gothenburg, Sweden; 4 Health Technology Assessment Centre Region Västra Götaland, Sahlgrenska University Hospital, Gothenburg, Sweden; 5 School of Medical Sciences, Faculty of Medicine and Health, Örebro University, Örebro, Sweden; 6 Department of Surgical Sciences, Uppsala University Hospital, Uppsala, Sweden; 7 Central Hospital in Karlstad, Region Varmland, Karlstad, Sweden; 8 Department of Vascular Surgery, Sahlgrenska University Hospital, Gothenburg, Sweden

**Keywords:** Health status, Nordic walking, Patient reported outcome measure, Peripheral arterial disease, Questionnaires

## Abstract

**Background:**

Supervised exercise is an integral part of the recommended first-line treatment for patients with intermittent claudication (IC). By reflecting the patients’ perspectives, patient-reported outcome measurements provide additional knowledge to the biomedical endpoints and are important outcomes to include when evaluating exercise interventions in patients with IC. We aimed to evaluate the one-year impact of three strategies: unsupervised Nordic pole walk advice (WA), WA + six months of home-based structured exercise (HSEP) or WA + six months of hospital-based supervised exercise (SEP) on health-related quality of life and patient-reported physical function in patients with IC.

**Methods:**

This secondary exploratory analysis of a multi-center, randomized clinical trial compared three exercise strategies. The primary outcome of the secondary analysis was the one-year change in the 36-Item Short-Form (SF-36). Secondary outcomes were three- and six-months SF-36 changes alongside three, six- and 12-months changes in the disease-specific Vascular Quality of Life instrument (VascuQoL) and the Patient-Specific Functional Scale (PSFS). The Kruskal–Wallis test with Bonferroni-adjusted post-hoc tests were used for between-group comparisons. Effect size calculations were used to describe the size of observed treatment effects, and the clinical meaningfulness of observed changes in the VascuQoL summary score at one year was studied using established minimally important difference (MID) thresholds.

**Results:**

A total of 166 patients with IC, mean age: 72.1 (SD 7.4) years, 41% women, were randomized. No significant between-group differences were observed over time for the SF-36 or the PSFS scores whereas some significant between-group differences were observed in the VascuQoL domain and summary scores over time, favoring SEP and/or HSEP over WA. The observed SF-36 and VascuQoL domain and summary score effect sizes were small to moderate, and many domain score effect sizes also remained unchanged over time. A significantly higher proportion of the patients in the SEP group reached the VascuQoL summary score MID of improvement in one year.

**Conclusion:**

Clinically important improvements were observed in SEP using the VascuQoL, while we did not observe any significant between-group differences using the SF-36. Whereas effect sizes for the observed changes over time were generally small, a significantly higher proportion of patients in SEP reached the VascuQoL MID of improvement.

**Trial registration:**

NCT02341716, January 19, 2015 (retrospectively registered).

**Supplementary Information:**

The online version contains supplementary material available at 10.1186/s12955-023-02198-8.

## Background

Patients with lower limb peripheral arterial occlusive disease (PAOD) and symptoms of intermittent claudication (IC) often experience functional impairments [1] and reduced health-related quality of life (HRQoL) mainly in HRQoL aspects of physical functioning and pain as compared with healthy individuals [2]. Supervised exercise is a guideline-recommended first-line treatment in IC that can improve functional status, HRQoL and reduce leg symptoms [3, 4], and furthermore may reduce the overall cardiovascular risk [5].

Patient-reported outcome measurements (PROMs) is an umbrella term for self-rating instruments that measure concepts such as health status, HRQoL, well-being, patient satisfaction, patient symptoms and functioning [6]. PROMs capture patient’s own opinions on important areas affected by the disease, such as pain, everyday functional limitations and social and emotional consequences of living with the disease. In clinical research, PROMs play an increasingly significant role as complementary endpoints by providing additional value and information to the more traditional biomedical endpoints [7].

Living with IC can impact many aspects of daily life [8] and an additional goal when using PROMs in IC can be to evaluate whether a certain exercise intervention that leads to an objectively assessed increased walking distance also translates to daily life improvements as experienced by the patient [9]. Limited by overall low quality of evidence, a meta-analysis of studies in patients with IC indicated some improvements in generic HRQoL parameters that favored supervised exercise over walk advice while no clear differences in generic HRQoL or patient-reported functional impairment between supervised exercise and home-based exercise could be confirmed [10]. Additionally, the impact of other modes of exercise than walking on PROM endpoints in patients with IC remains unclear [11].

In the recently published randomized clinical SUNFIT trial (Supervised or UNsupervised exercise training For Intermittent claudicaTion), we determined the comparative effectiveness of three different treatment strategies on walking performance and lower limb muscle endurance [12]. All patients received best medical treatment, free Nordic poles and an unsupervised walk advice (WA) to regularly perform Nordic pole walking. In addition to WA, the two other strategies included six months of a home-based structured exercise program (HSEP) or a hospital-based supervised exercise program (SEP). Our results demonstrated that HSEP was non-inferior to SEP whereas no significant differences were observed between SEP, HSEP and the unsupervised WA strategy alone at one-year, neither with regard to the six-minute walk test (6MWT) nor with regard lower limb muscle endurance tests [12]. In this secondary exploratory analysis of the SUNFIT trial, we aimed to evaluate the one year impact of WA, WA + HSEP and WA + SEP on generic and disease-specific HRQoL and self-reported physical function in patients with IC.

## Methods

**Fig. 1:**
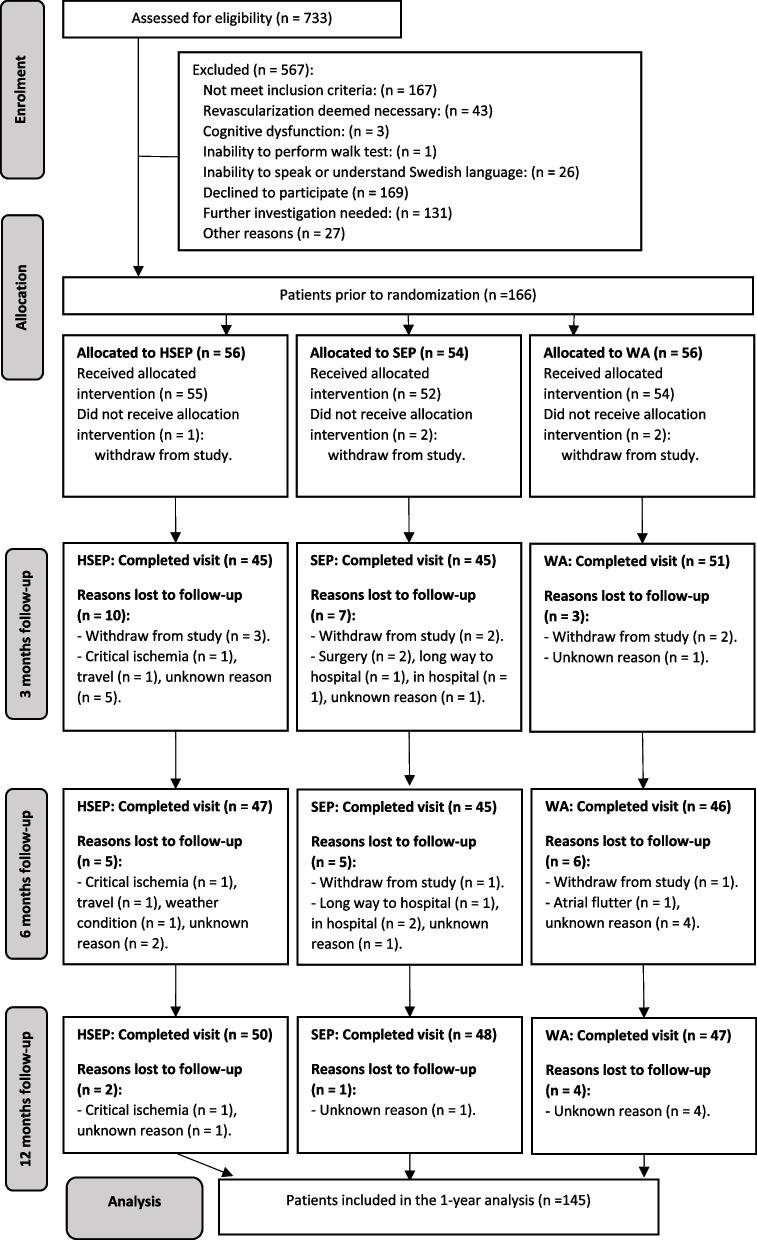
CONSORT study flow chart, presented by treatment strategy. HSEP, home-based structured supervised exercise program. SEP, hospital-based supervised exercise program, WA, walk advice

The key study characteristics of the SUNFIT trial are summarized in Table 1 and a Consolidated Standards of Reporting Trials (CONSORT) study participation and follow-up flow chart is presented in Fig. 1, while further study details have been reported elsewhere [12]. Informed, written consent was obtained from all participants. The study conduct followed the principles in the Declaration of Helsinki and the study protocol was approved by the Regional Ethical Review Board in Gothenburg (entry no. 349–14, T789-16).

**Table 1:** Details of design and methods in the SUNFIT trial

**Characteristics**	
Study design	Prospective, multi-center, randomized clinical trial with three treatment arms; with a confirmatory intention-to-treat non-inferiority analysis of the main endpoint (6MWT) and exploratory superiority testing of secondary trial endpoints
ClinicalTrials.gov identifier	NCT02341716
Inclusion criteria	Established mild to severe IC for > 6 months, confirmed to be of vascular origin, with an ABI of less than 0.9 and/or a post-exercise ABI drop of ≥ 30%
Exclusion criteria	- Previous revascularization for IC performed within three months - Revascularization within 12 months deemed necessary by the vascular surgeon - Cognitive dysfunction - Inability to perform the 6MWT - Inability to speak or understand the Swedish language
Recruitment	Patients were recruited among patients referred for evaluation regarding revascularization to the vascular surgery outpatient clinics at the Departments of Vascular Surgery at Sahlgrenska University Hospital, the Region Hospital of Karlstad and Södra Älvsborg Hospital, Sweden
Recruitment period	September 2014 to January 2018
Randomization and blinding	Randomization using an adaptive stratified computerized randomization procedure (multifactorial minimization) [13] Endpoint evaluators were blinded to group affiliations
Follow up visits	At three, six and 12 months for endpoint evaluations at the Physiotherapy Department; and a revisit to the vascular surgeon at 12 months
Classification of exercise adherence	Full adherence: attendance at ≥ 80% of the exercise sessions at recommended exercise intensity for ≥ 30 min per session Partial adherence: attendance at ≥ 20 to < 80% of exercise sessions, irrespective of exercise intensity Non-adherence: attendance at < 20% of exercise sessions

*6MWT* Six-minute walk test, *IC* Intermittent claudication, *ABI* Ankle brachial index

### Study procedure

At baseline, all patients received verbal and written disease information, best medical treatment, and a free pair of walking poles [12]. The unsupervised WA was provided by the vascular surgeon or vascular nurses and included advice on IC limb symptom-inducing Nordic pole walking sessions for at least 30 min three times weekly. Guideline-recommended secondary prevention medications [4] were instituted or enhanced for all patients unless contraindicated, and a smoking cessation program was offered to smokers. Two teams of physiotherapists participated in this study; one team was responsible for the evaluation of study outcomes (blinded to group affiliations) and the other team provided HSEP and SEP. Logbooks were used for measuring exercise adherence (0–6 months) in HSEP and SEP and the definition of exercise adherence is described in Table 1. After randomization, all patients visited the evaluating physiotherapist for baseline PROMs assessments. The PROMs were completed by the patients at the subsequent outcome evaluation visits that were arranged at three, six and 12 months.

### Treatment strategies

#### Walk advice (WA)

This strategy included best medical treatment and the unsupervised WA of Nordic pole walking for at least 30 min, three times weekly.

#### Home-based structured exercise program (HSEP)

This strategy included the WA and an additional HSEP with aerobic walking exercises and resistance exercises. Patients were instructed to perform three 50-min exercise sessions weekly at home, for a total program duration of one year. The first six months was structured, with feedback from the physiotherapist by biweekly phone calls and two face-to-face visits. For the remaining six months, patients were instructed to continue with the prescribed HSEP but without further feedback.

#### Hospital-based supervised exercise program (SEP)

This strategy included the WA and an additional SEP. The SEP comprised the same exercise content and dose as HSEP but was undertaken as group sessions at the hospital under supervision from the physiotherapist (0–6 months). In accordance with HSEP, for the remaining six months, patients were instructed to continue with the prescribed program at home without further supervision or feedback.

### Patient-reported outcome measurements

#### The 36-item Short-Form (SF-36)

The SF-36 is a valid and reliable generic questionnaire that includes 36 items covering different aspects of HRQoL in eight different domains: PF = physical functioning; RP = role physical; BP = bodily pain; GH = general health; VT = vitality; SF = social functioning; RE = role emotional and MH = mental health. The results are also presented in two summary components scores: the physical component summary (PCS) and the mental component summary (MCS) [14–16]. The SF-36 is considered reliable among Swedish patients with IC [17].

#### The Vascular Quality of life (VascuQoL)

The VascuQoL is a disease-specific HRQoL questionnaire, designed and frequently used to evaluate treatment effects in patients with IC. The VascuQoL includes 25 items, categorized into five domains: Pain, Symptoms, Activities, Emotional, and Social. The VascuQoL Summary score is the sum of all items mean scores, divided by 25. The item responses range from 1 (worst HRQoL) to 7 (best HRQoL) [18]. The VascuQoL is valid, reliable and responsive among patients with IC within a Swedish context [19]. The minimally important difference (MID) of the VascuQoL Summary score among patients with IC has been calculated previously using an anchor-based approach, with thresholds ≥ 0.87 for improvement and ≤ 0.23 for deterioration [20]. The same thresholds were also used when analyzing MIDs for the VascuQoL in this study.

#### The Patient-Specific Functional Scale (PSFS)

Physical function is defined as the capacity of an individual to carry out the physical activities of daily living. Physical function reflects motor function and control, physical fitness, and habitual physical activity [21]. The Patient-Specific Functional Scale (PSFS) questionnaire measures change in physical function in response to treatment and is applicable to different health conditions and interventions. Patients are asked to nominate up to five physical activities which they have difficulty performing due to their condition, and then rate their functional limitations during the nominated activities. The difficulty of each activity is rated on a Likert scale ranging from 0 (being unable to perform the activity) to 10 (being able to perform the activity without difficulty) [22]. In this study, patients were asked to nominate and rate ≥ three activities at baseline and then evaluate the same activities at follow-up. In order to overview the patient-selected activities, physical activity can be categorized into four domains: a) leisure-time, b) work-related, c) household/domestic or self-care d) transportation [23]. The PSFS is reliable, valid, and responsive to change in different musculoskeletal conditions [22, 24] and older adults [25] but has not been validated in IC.

### Statistical methods

All patients that attended follow-up and completed the SF-36, VascuQoL and PSFS questionnaires were included in this exploratory superiority analysis comparing the three treatment strategies (WA, WA + HSEP and WA + SEP). For all outcomes, the full data set was used without any imputation and all analyses were based on the initial treatment assignment. The principal outcome measure in this secondary analysis was change in the SF-36 scores from baseline to one year. Other outcomes of interest were the change in the SF-36 scores from baseline to three and six months, respectively, and the change in the VascuQoL and the PSFS scores from baseline to three, six and 12 months. The primary analysis in the SUNFIT trial had a non-inferiority design, and the sample size was based on the primary hypothesis that HSEP would be non-inferior to SEP regarding 6MWT change, presented in detail elsewhere [12]. Nominal data are presented as absolute numbers and percentages while normally distributed continuous data are presented as the mean with one standard deviation (SD) and non-normally distributed continuous data and ordinal data as the median and interquartile range (IQR). For intergroup comparisons, the non-parametric Kruskal–Wallis test was used and observed significant differences between groups were further analyzed with pairwise Mann–Whitney U post-hoc tests, adjusted by the Bonferroni correction for multiple tests. The standard SF-36 scoring algorithms were used, and each item score was coded, recalibrated, summed, and transformed into a scale from 0–100, where a higher score indicates better health [26]. The overall internal missing of SF-36 and VascuQoL items were 1%. No item imputation procedures were used. Internal missing data for the SF-36 were handled as follows; if at least one-half of the items in a scale were answered the domain was calculated whereas the PCS or MCS were not calculated if one or more domains were missing. Similarly, if one or more VascuQoL items were missing no domain score was calculated. To compare the observed proportions of patients reaching the MID thresholds for the VascuQoL Summary score for improved, unchanged and deteriorated health status [20] at one year, a Chi-Square test for independence (3 by 3) was used. In a sensitivity analysis, all analyses were also done following the removal of patients that underwent lower limb revascularization during the follow-up period. Observed significant results were further analyzed with post-hoc tests, adjusted by the Bonferroni correction for multiple tests. Effect sizes were calculated by dividing the observed mean difference in the PROM values at three, six and 12 months respectively with the corresponding baseline standard deviation. No further between group statistical testing was undertaken on effect sizes. Cohen’s criteria for interpreting effect size calculations were applied (small = 0.2 to < 0.5; moderate = 0.5 to < 0.8; large > 0.8) [27]. Statistical significance was assumed at *p* < 0.05. Statistical analysis was performed using SPSS® version 25 (IBM, Armonk, NY, USA).

## Results

### Study population

Out of the 166 randomized patients, five patients did not receive the allocated intervention (HSEP *n* = 1, SEP *n* = 2 and WA *n* = 2) and a further nine patients subsequently withdrew from the study. At one year, seven patients (HSEP *n* = 2, SEP *n* = 1 and WA *n* = 4) were lost to follow-up, leaving a total of 145 (87%) patients for this analysis (Fig. 1). The baseline characteristics of the study population are given in Table 2 and no significant between-group differences were observed at baseline in terms of demographics, risk factors or comorbidities. A total of eight patients underwent lower limb revascularization during the study period due to deterioration of lower limb symptomatology (HSEP *n* = 4 and SEP *n* = 4). Nine (24%) and 14 patients (26%) were fully adherent and 27 (71%) and 26 (48%) in HSEP and SEP respectively were partially adherent to the six months exercise programs.

**Table 2:** Baseline descriptive (*n* = 166), presented by treatment strategy

**Variable**	**HSEP** *n* = 56	**SEP** *n* = 54	**WA** *n* = 56
**Gender**, female, n (%)	21 (37.5)	23 (42.6)	24 (42.9)
**Age**, years, mean (SD)	71.8 (6.5)	72.2 (7.5)	72.5 (8.2)
**Ankle-brachial index**, mean (SD)	0.66 (0.27)	0.64 (0.18)	0.67 (0.21)
**Affected leg**, n (%)			
Right	15 (26.8)	13 (24.1)	16 (28.6)
Left	14 (25.0)	12 (22.2)	17 (30.4)
Both	27 (48.2)	29 (53.7)	23 (41.1)
**Rutherford classification**, n (%)			
Mild claudication	8 (14.3)	11 (20.4)	11 (19.6)
Moderate claudication	34 (60.7)	33 (61.1)	35 (62.5)
Severe claudication	14 (25.0)	10 (18.5)	10 (17.9)
**Smoking**, n (%)			
Yes	15 (26.8)	16 (29.6)	16 (28.6)
Earlier	37 (66.1)	33 (61.1)	34 (60.7)
Never	4 (7.1)	5 (9.3)	6 (10.7)
**Physical activity**^**a**^, yes, n (%)	16 (28.6)	16 (29.6)	21 (37.5)
**Co-morbidity**, n (%)			
Heart disease^b^	18 (32.1)	18 (33.3)	17 (30.4)
Chronic obstructive pulmonary disease	8 (14.3)	8 (14.8)	7 (12.5)
Diabetes mellitus	19 (33.9)	14 (25.9)	16 (28.6)

No significant differences were observed between the three groups at baseline

*HSEP* Home-based structured exercise program, *SEP* Hospital-based supervised exercise program, *WA* Walk advice

^a^Physical activity was defined as walking for at least 30 min, three times weekly

^b^Heart disease included a diagnosis of chronic heart failure, stable angina pectoris or previous acute coronary syndrome

### Patient-reported outcome measurements

At baseline, no significant between-group differences were observed for any of the PROM questionnaires.

#### The 36-Item Short-Form

No statistically significant between-group differences were observed for the SF36 domain scores, PCS or MCS over time. Baseline data and observed changes at three, six and 12 months in the SF-36 score data are presented by group in Table 3.

**Table 3:** The SF-36 and the VascuQol, presented by group and over time

	HSEP				SEP				WA				Between group *P*-value		
		Change at:				Change at:				Change at:					
SF-36															
Domains	Base-line	3 months	6 months	12 months	Base-line	3 months	6 months	12 months	Base-line	3 months	6 months	12 months	0–3 months	0–6 months	0–1 year
PF	50.0 (35.0)	5.0 (12.1)	5.0 (20.0)	5.0 (17.6)	55.0 (30.0)	0.0 (20.0)	10.0 (16.8)	10.0 (20.0)	52.5 (27.5)	5.0 (20.0)	2.8 (20.0)	10.0 (15.0)	.724	.354	.391
RP	50.0 (100)	0.0 (25.0)	0.0 (25.0)	0.0 (25.0)	25.0 (100)	0.0 (12.5)	0.0 (47.9)	0.0 (25.0)	25.0 (81.3)	0.0 (25.0)	0.0 (50.0)	0.0 (66.7)	.569	.410	.941
BP	41.0 (11.0)	10.0 (20.0)	10.0 (29.0)	10.0 (23.8)	41.0 (25.5)	10.0 (24.0)	0.0 (21.5)	10.0 (26.0)	51.0 (26.0)	0.0 (21.0)	5.0 (12.5)	0.0 (31.3)	.107	.920	.149
GH	57.0 (26.5)	0.0 (19.3)	5.0 (23.0)	5.0 (20.0)	57.0 (27.0)	5.0 (18.8)	4.0 (20.0)	2.0 (20.0)	57.0 (25.0)	0.0 (15.7)	0.0 (20.0)	5.0 (17.1)	.223	.271	.855
VT	60.0 (30.0)	5.0 (15.0)	5.0 (20.0)	0.0 (15.0)	57.5 (26.3)	2.5 (18.8)	2.5 (25.0)	5.0 (15.8)	60.0 (32.5)	0.0 (10.0)	0.0 (15.0)	0.0 (20.0)	.735	.115	.525
SF	87.5 (37.5)	0.0 (12.5)	0.0 (12.5)	0.0 (21.9)	81.3 (37.5)	0.0 (25.0)	0.0 (0.0)	0.0 (25.0)	87.5 (43.8)	0.0 (12.5)	0.0 (25.0)	0.0 (15.6)	.282	.909	.329
RE	66.7 (100)	0.0 (33.3)	0.0 (8.3)	0.0 (33.3)	66.7 (100)	0.0 (33.3)	0.0 (33.3)	0.0 (33.3)	66.7 (75.0)	0.0 (33.3)	0.0 (66.7)	0.0 (33.3)	.963	.755	.627
MH	76.0 (28.0)	4.0 (14.0)	0.0 (20.0)	0.0 (16.0)	80.0 (32.0)	2.0 (15.0)	2.0 (16.0)	8.0 (17.0)	84.0 (24.0)	0.0 (16.5)	2.0 (12.0)	0.0 (13.0)	.096	.751	.133
PCS	37.0 (14.7)	0.9 (6.6)	2.1 (8.8)	3.3 (7.4)	35.2 (13.5)	2.2 (9.9)	3.5 (10.4)	3.4 (7.9)	35.4 (15.2)	0.6 (7.6)	0.1 (6.7)	2.4 (8.0)	.914	.212	.720
MCS	49.5 (17.7)	1.6 (9.2)	1.3 (8.5)	0.0 (12.0)	51.0 (21.2)	0.8 (13.0)	-0.1 (8.5)	2.2 (9.2)	51.0 (19.1)	0.9 (12.0)	-1.0 (10.5)	0.6 (10.2)	.694	.555	.294
VascuQol															
n	55	44	47	50	52	45	43	47	54	50	46	47			
Activity	4.31 (1.44)	0.50 (0.69)	0.25 (0.88)	0.50 (1.13)	4.38 (1.00)	0.50 (1.13)	0.56 (1.28)	0.50 (1.41)	4.50 (1.69)	0.25 (0.88)	0.25 (1.03)	0.13 (0.88)	.076	.390	.169
Symptom	4.25 (1.50)	0.50 (1.25)	0.25 (1.00)	0.38 (1.25)	3.75 (2.00)	0.00 (1.50)	0.50 (1.56)	0.25 (1.06)	4.00 (1.50)	0.25 (1.25)	0.00 (1.44)	0.25 (1.44)	.186	.511	.599
Pain	4.75 (1.50)	0.38 (0.94)	0.25 (1.00)	0.50 (1.38)	4.13 (2.00)	0.25 (1.13)	0.75 (1.00)	0.75 (1.50)	4.75 (1.19)	0.25 (1.00)	0.13 (0.75)	0.50 (1.00)	.072	.002	.057
Emotional	5.00 (1.64)	0.43 (1.00)	0.29 (1.07)	0.43 (1.43)	5.29 (1.57)	0.57 (1.50)	0.57 (1.36)	0.43 (1.14)	5.71 (1.86)	0.14 (0.75)	0.07 (0.79)	0.29 (0.86)	.010	.065	.245
Social	5.00 (2.50)	0.00 (1.00)	0.00 (1.00)	0.25 (1.50)	4.50 (2.38)	1.00 (1.50)	0.50 (1.00)	1.00 (1.50)	5.25 (2.50)	0.50 (1.50)	0.00 (1.00)	0.20 (0.80)	.080	.090	.040
Summary score	4.70 (1.25)	0.44 (0.78)	0.36 (0.68)	0.38 (0.89)	4.42 (1.28)	0.48 (0.94)	0.60 (1.08)	0.60 (0.96)	4.84 (1.24)	0.12 (0.68)	0.18 (0.56)	0.20 (0.80)	.008	.018	.060

Data are presented as the median and interquartile range (IQR)

*HSEP* Home-based structured supervised exercise program. *SEP* Hospital-based supervised exercise program. *WA* Walk advice. Between group differences are calculated with the Kruskal Wallis Test. *PF* Physical functioning, *RP* Role physical, *BP* Bodily pain, *MH* Mental health, *RE* Role emotional, *SF* Social functioning, *VT* Vitality and *GH* General health, *PCS* Physical component summary, *MCS* Mental component summary. Higher scores indicate better HRQoL

#### The Vascular Quality of Life

Baseline data and changes at three, six and 12 months in the VascuQoL score data are presented by group in Table 3. At one year, a significant between-group difference was found in the Social domain but the post hoc analysis revealed no significant between-group differences. At three months, in the Emotional domain, a significant between-group difference was observed, in favor of SEP versus WA (*p* = 0.035) and in favor of HSEP versus WA (*p* = 0.020). At three months, a significant between-group difference was also observed in the Summary score in favor of HSEP versus WA (*p* = 0.023) and in favor of SEP versus WA (*p* = 0.023). At six months, significant between-group difference was found in the Pain domain in favor of SEP versus WA (*p* = 0.002) and in favor of SEP versus HSEP (*p* = 0.035) and in the Summary score, in favor of SEP versus WA (*p* = 0.014).

**Fig. 2:**
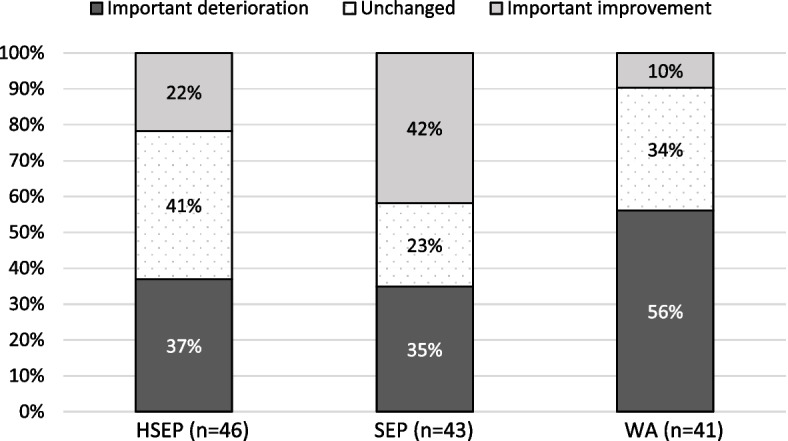
Proportion of patients reaching a threshold for important deterioration or improvement in the VascuQoL Summary score change from baseline to 1 year*.* HSEP, home-based structured supervised exercise program. SEP, hospital-based supervised exercise program, WA, walk advice

Figure 2 presents the proportion of patients reaching the MID thresholds for important improvement, unchanged score or important deterioration at one year, as determined by the VascuQoL summary score. In SEP, 42% of the patients reached the threshold for improvement as compared with 22% in HSEP and 10% in WA. A significant result (*p* = 0.007) was observed when comparing the between-groups proportions of patients reaching the MID thresholds of improvement, unchanged and deterioration, and the post-hoc analysis showed that a significantly higher proportion of patients in SEP reached the MID for improvement (p < 0.001). In a further sensitivity analysis, this observation also remained after removal of patients (*n* = 8) that were revascularized during follow up.

#### Effect sizes for the 36-Item Short-Form and the Vascular Quality of Life questionnaires

Table 4 details the observed effect sizes by category (i.e., small, moderate and large) and by treatment strategy over time, for the VascuQoL and the SF-36 instruments and, corresponding numerical values of the effect sizes are presented in Additional file 1: Appendix 1. In general, the observed treatment effects were small to moderate, and many domain score effect sizes remained unchanged over time. None of the treatment strategies led to a large treatment effect in any of the studied domain or summary scores.

**Table 4:** Effect size for the SF-36 and the VascuQoL at three, six and 12 months

	HSEP			SEP			WA		
Variable	**3 months**	**6 months**	**12 months**	**3 months**	**6 months**	**12 months**	**3 months**	**6 months**	**12 months**
PF	Small	Small	Small	Small	Small	Moderate	Small	Small	Moderate
RP	Small		Small		Small	Small			Small
BP	Small	Small	Moderate	Small	Small	Moderate		Small	
GH		Small		Small					
VT						Small			
SF									Small
RE	Small	Small				Small	Small		Small
MH	Small					Small			
PCS	Small	Small	Small	Small	Small	Small	Small	Small	Small
MCS									
Activity	Moderate	Small	Small	Moderate	Small	Moderate		Small	Small
Symptom	Small		Small		Small	Small			
Pain	Small	Small	Small	Moderate	Moderate	Moderate			Small
Emotional	Small	Small	Small	Small	Small	Small			
Social	Small			Small	Small	Moderate	Small	Small	Small
Summary Score	Small	Small	Small	Small	Small	Moderate			Small

Effect size: small = 0.2 to < 0.5; moderate = 0.5 to < 0.8; large > 0.8. Empty table panel indicate no effect size (unchanged)

*HSEP* Home-based structured supervised exercise program. *SEP* hospital-based supervised exercise program, *WA* Walk advice. *PF* Physical functioning, *RP* Role physical, *BP* Bodily pain, *MH* Mental health, *RE* Role emotional, *SF* Social functioning, *VT* Vitality and *GH* General health, *PCS* Physical component summary, *MCS* Mental component summary

#### The Patient-Specific Functional Scale

**Fig. 3:**
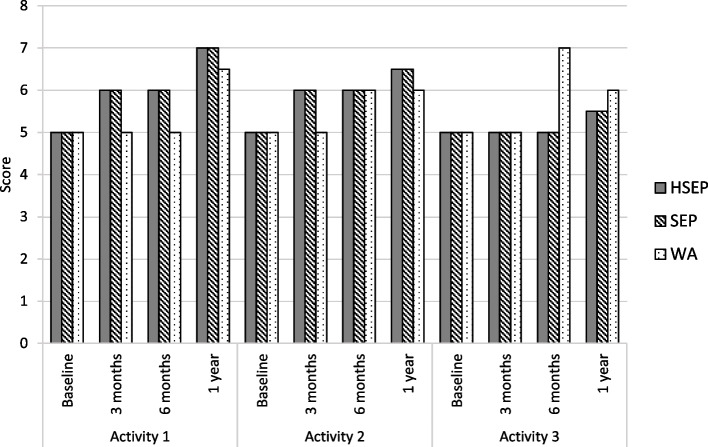
Median values in the three activities in the Patient-Specific Functional Scale, presented by treatment strategy, over time. HSEP, home-based structured supervised exercise program. SEP, hospital-based supervised exercise program, WA, walk advice

The patient-selected physical activities are described, categorized and presented for the entire study population in Table 5. Transport-related activities such as walking, walking up stairs and hills were the most patient-selected activities. Figure 3 shows the PSFS scores at baseline and over time, presented by treatment arm and for the three nominated activities. No between-group differences were observed in the PSFS scores for any of the three activities over time.

**Table 5:** The patient-selected activities in the Patient-Specific Functional Scale, categorized into the four domains of physical activity and presented for the entire study population

	**Activity 1**	**Activity 2**	**Activity 3**
Leisure-time physical activities, e.g., golf, tennis, bicycling, dance, visit restaurants, visit grandchildren, shopping. n (%)	8 (4.8)	16 (9.6)	20 (12.0)
Work-related activities, n (%)	5 (3.0)	1 (0.6)	0
Household/domestic/self-care activities, e.g., rise from sitting, pick up things from the floor, gardening, n (%)	17 (10.2)	24 (14.5)	31 (18.7)
Transport-related activities, e.g., walking activities, walking up stairs and hills, n (%)	131 (78.9)	109 (65.7)	73 (44.0)
Missing, n (%)	5 (3.0)	16 (9.6)	42 (25.3)
Sum of patients per activity, n	161^a^	150	124

Number of patients presented by activity

^a^Five out of 166 patients did not receive allocated intervention

## Discussion

While the previously reported primary endpoint results in the SUNFIT trial (6MWT) did not differ between treatment strategies [12], this secondary exploratory analysis uncovered significant HRQoL differences as measured by the disease-specific VascuQoL instrument, which indicated that hospital-based supervised exercise therapy might be superior to the other two studied exercise strategies in terms of improvement in disease-specific HRQoL. These findings are important as clinically meaningful HRQoL improvement is probably the most important overall goal when offering treatments that target lower limb symptoms in patients with intermittent claudication [3]. By contrast, we did not observe any significant differences over time when analyzing the SF-36 scores from baseline to one year. The SF-36 results in our study stand in contrast to the Cochrane Report by Hageman et al.[10] that indicated a benefit of SEP over WA for the SF-36 physical functioning, pain, and PCS after nine months and one year of follow-up. Those results were also supported by the meta-analysis by Parmenter et al. [11] which demonstrated that supervised walking exercise significantly improved the SF-36 PCS when compared with usual care, with or without exercise advice. Overall, current interpretations of the effects on PROM in response to different exercise interventions in IC are supported by low to moderate quality of evidence, mainly limited by the small number of studies and participants within trials [10, 28]. Moreover, most studies have mainly evaluated the effects of treadmill-based exercise programs whereas the effects of other exercise modalities remain poorly studied [10]. Clearly, more studies that investigate various optimized exercise strategies are needed to determine the maximally achievable HRQoL treatment effects in patients with IC.

At three months, a significant difference was observed, in favor of both SEP and HSEP versus WA, in the VascuQoL Emotional domain as well as in the overall Summary score. In the VascuQoL Summary score, this significant difference in favor of SEP versus WA also remained at six months. Additionally at six months, a significant difference was observed in the VascuQoL Pain domain, in favor of SEP as compared with the other offered treatment strategies. While the observed effect sizes were generally small to moderate, these exploratory findings might still be related to important treatment differentials that were not evident when assessed by objective walking tests such as maximal- and pain-free walking distance as reported in the primary analysis of the SUNFIT trial [12]. By integrating PROMs in clinical trials, a better understanding of patient- and treatment-related factors associated with improved or deteriorated health status over time can be achieved [29]. Therefore, our findings may provide an example of the importance of integrating PROMs alongside objective endpoints in clinical trials.

At three and six months, we observed significant between-group results regarding some VascuQoL domain and summary items favoring SEP and HSEP as compared with WA. In addition to the exercise performed in SEP and HSEP, the potential benefits of the exercise encouragement, support and feedback given by the physiotherapists [30], may have contributed to these results. In SEP, also the support/interaction with other patients with IC may have contributed to these findings [30]. Overall, social and emotional support are important contributing factors to health [31]. Moreover, to the best of our knowledge this is the first randomized clinical trial evaluating SEP, HSEP and WA with the VascuQoL questionnaire. A previous network meta-analysis of RCTs that compared the outcomes of different IC treatments (cilostazol, endovascular revascularization, SEP, HSEP and control treatment) indicated that the VascuQoL was used as an outcome measure in only six of the 46 included RCTs, and these six trials evaluated SEP versus endovascular revascularization (*n* = 4), and cilostazol (*n* = 1) or endovascular revascularization compared with control (*n* = 1) [32]. In a non-randomized study by Fakhry et al*. *[33], no VascuQoL score differences between HSEP and SEP at six and 12 months were observed.

A significantly higher proportion of patients in the SEP group (compared with HSEP and WA) reached the VascuQoL summary score MID for improvement at the one-year follow-up visit. The MID thresholds calculated by Conjin et al. [20] that were used in this explorative analysis suggested a MID of important deterioration at an estimated positive value of 0.23 scores. The use of MID rather than raw score changes over time is important since if we had only used the latter this would have resulted in a false impression of improvement across all three study groups. Additionally, the majority (56%) of the patients in WA reached the MID of important deterioration at one year, as compared with 37% in HSEP and 35% in SEP (not significant between groups). It seems reasonably likely that the patients in WA reaching a MID of deterioration were less likely to follow the unsupervised walk advice of Nordic pole walking. However, as the adherence to the walk advice was not assessed (to keep the WA truly unsupervised) this remains purely speculative.

Moreover, it is important to consider the context in which these thresholds were derived. Compared with our one-year result, Conjin and colleagues had a shorter follow-up period of three to four months. Also, the IC treatment offered differed in the studied population and included SEP, percutaneous transluminal angioplasty (PTA), and surgical revascularization while some patients only received best medical treatment (antiplatelet drug and a statin, unsupervised walk advice and advice on lifestyle changes). However, the MID values can be applied irrespective of sample size and are thus useful in both individual care and research settings [20].

The fact that the SF36 is a generic PROM that provides a wider HRQoL measure than PAOD-specific PROMs [34] may be a possible explanation of the more frequently observed effect size changes over time for the VascuQoL as compared with the SF-36 in our study. For example, the VascuQoL provides several additional themes for symptoms (e.g., mobility and walking ability) and impact on physical functioning including daily activities and exercise as compared with the themes in the SF-36 [35]. As compared with other generic PROMs (e.g., EuroQol 5-dimensions; EQ-5D), the SF-36 probably has the most comprehensively evaluated psychometric properties in IC patients [34] and we suggest that future IC-research use both generic and disease-specific PROMs.

To the best of our knowledge, this is the first study evaluating exercise programs with the PSFS in patients with IC. The impact of exercise has previously been evaluated with the PSFS in patients with low back pain [36] and in patients with knee osteoarthritis [37]. For the PSFS, floor and ceiling effects have been poorly investigated [24]. This implies that if patients select very difficult activities as their most impaired activities, the probability of reporting improvement of these activities may be limited [38]. On the other hand, the activities in the PSFS are self-selected, and thus more likely to be important to the patient.

There are many different questionnaires evaluating HRQoL in patients with IC and the use of these tools in a research context is inconsistent and non-standardized [29]. A strength of our study is the inclusion of both generic and disease specific HRQoL PROMs. Additional strengths of this study are the randomized design and the blinding of endpoint evaluators which generated statistically similar groups at baseline and avoided observer bias. An important study limitation is that the sample size estimation was based on the 6MWT rather than PROM outcomes. Therefore, this study may have been underpowered for the performed analysis, and therefore the reported results must be regarded as exploratory rather than confirmatory. This study was further limited by relatively poor exercise adherence to SEP and HSEP. Whereas most participants were partly or fully adherent with the offered exercise programs, only a few patients were considered as fully exercise adherent [12]. Another limitation of our study is that few patients were able to nominate two or three activities in the PSFS, resulting in less power to this calculation and therefore a non-negligible risk of a type II error for this particular endpoint.

## Conclusion

Potentially important treatment differentials were revealed when analyzing the disease-specific VascuQoL, while we did not observe any significant between-group differences for the SF-36. Whereas effect sizes for the observed changes in the SF-36 and the VascuQoL over time were generally small, a significantly higher proportion of patients in the SEP group reached the minimally important difference threshold for improvement in the VascuQoL Summary score at one year. Our study demonstrates the importance of including disease-specific instruments when evaluating PROM endpoints in patients with intermittent claudication and also emphasize the potential importance of integrating PROMs alongside more traditional endpoints in clinical trials.

### Supplementary Information

**Additional file 1:** **Appendix 1.** Effect size presented by numerical values for the SF-36 and the VascuQoL at three, six and 12 months.

## Data Availability

The dataset generated and analyzed during the current study is not available due to ethical considerations and General Data Protection Regulation (GDPR). Processed summary data are however available on reasonable request. The primary article for this randomized clinical trial: (DOI number: 10.1093/eurjcn/zvac070.)

## Abbreviations

6MWT: Six-minute walk test
ABI: Ankle brachial index
HRQOL: Health-related Quality of life
HSEP: Home-based structured exercise program
IC: Intermittent claudication
MID: Minimally important difference
RCT: Randomized clinical trial
SEP: Hospital-based supervised exercise program
SF-36: 36-Item Short-Form
SUNFIT: Supervised or UNsupervised exercise training For Intermittent claudication
PROM: Patient-reported outcome measurement
PSFS: Patient-specific functional scale
VascuQoL: Vascular Quality of life
WA: Walk advice
