# Supplementary material for: Impact of walk advice alone or in combination with supervised or home-based structured exercise on patient-reported physical function and generic and disease-specific health related quality of life in patients with intermittent claudication, a secondary analysis in a randomized clinical trial
Source: Health Qual Life Outcomes. 2023 Oct 23;21:114. doi: 10.1186/s12955-023-02198-8 (PMC10594797; doi:10.1186/s12955-023-02198-8)
Supplement: Supplementary file 1 — Additional file 1: Appendix 1. Effect size presented by numerical values for the SF-36 and the VascuQoL at three, six and 12 months. [file 12955_2023_2198_MOESM1_ESM.pdf]

Appendix 1. Effect size presented by numerical values for the SF-36 and the VascuQoL at three, six and 12 months.

	HSEP			SEP			WA		
**Variable**	**3 months**	**6 months**	**12 months**	**3 months**	**6 months**	**12 months**	**3 months**	**6 months**	**12 months**
**PF**	0.27	0.27	0.39	0.24	0.48	0.54	0.37	0.31	0.51
**RP**	0.22	0.12	0.21	0.13	0.31	0.30	0.19	0.02	0.32
**BP**	0.50	0.31	0.57	0.33	0.35	0.54	0.10	0.21	0.17
**GH**	-0.08	0.21	0.08	0.21	0.17	0.13	0.06	-0.06	0.14
**VT**	0.10	0.16	0.09	0.06	0.08	0.22	0.05	-0.18	0.08
**SF**	-0.01	0.04	-0.09	-0.07	0.01	0.02	0.16	0.05	0.21
**RE**	0.33	0.21	0.16	0.19	0.16	0.30	0.25	0.05	0.30
**MH**	0.29	0.00	0.03	0.13	0.06	0.25	0.01	0.03	0.11
**PCS**	0.22	0.31	0.45	0.22	0.43	0.43	0.24	0.21	0.37
**MCS**	0.19	0.07	-0.09	0.05	-0.05	0.09	0.10	-0.07	0.12
**Activity**	0.50	0.41	0.50	0.54	0.46	0.55	0.17	0.21	0.21
**Symptom**	0.38	0.19	0.38	0.16	0.30	0.27	0.10	0.09	0.17
**Pain**	0.41	0.22	0.41	0.54	0.61	0.66	0.14	0.13	0.30
**Emotional**	0.43	0.34	0.43	0.39	0.35	0.39	0.08	0.08	0.17
**Social**	0.18	0.10	0.18	0.50	0.42	0.55	0.23	0.28	0.21
**Summary Score**	0.50	0.35	0.46	0.47	0.50	0.56	0.14	0.16	0.24

Effect size: small=0.2 to <0.5; moderate=0.5 to <0.8; large >0.8. Empty table panel indicate no effect size (unchanged). HSEP, home-based structured supervised exercise program. SEP, hospital-based supervised exercise program, WA, walk advice. PF = physical functioning; RP= role physical; BP= bodily pain; MH= mental health; RE= role emotional; SF = social functioning; VT= vitality and GH= general health; PCS = physical component summary; MCS = mental component summary.
